# Ral A, via activating the mitotic checkpoint, sensitizes cells lacking a functional *Nf1* to apoptosis in the absence of protein kinase C

**DOI:** 10.18632/oncotarget.12607

**Published:** 2016-10-12

**Authors:** Suthakar Ganapathy, Johan B Fagman, Ling Shen, Tianqi Yu, Xiaodong Zhou, Wei Dai, Alexandros Makriyannis, Changyan Chen

**Affiliations:** ^1^ Center for Drug Discovery, Northeastern University, Boston, MA, USA; ^2^ The Institute of Clinic Sciences, Sahlgrenska Academy, Gothenburg, SE; ^3^ The First Affiliated Hospital of Nanchang University, Nanchang, China; ^4^ Department of Environmental Medicine, New York University, Tuxedo, NY, USA

**Keywords:** Nf1, Ral A, Chk1, mitotic catastrophe, apoptosis

## Abstract

*Nf1* mutations or deletions are suggested to underlie the tumor predisposition of NF1 (neurofibromatosis type 1) and few treatments are available for treating NF1 patients with advanced malignant tumors. Aberrant activation of Ras in Nf1-deficient conditions is responsible for the promotion of tumorigenesis in *NF1*. PKC is proven to be an important factor in supporting the viability of *Nf1*-defected cells, but the molecular mechanisms are not fully understood. In this study, we demonstrate that the inhibition of protein kinase C (PKC) by 1-O-Hexadecyl-2-O-methyl-rac-glycerol (HMG, a PKC inhibitor) preferentially sensitizes *Nf1*-defected cells to apoptosis, via triggering a persistent mitotic arrest. In this process, Ral A is activated. Subsequently, Chk1 is phosphorylated and translocated to the nucleus. Silencing Ral A significantly blocks Chk1 nuclear translocation and releases HMG-treated *Nf1*-deficient cells from mitotic arrest, resulting in the reduction of the magnitude of apoptosis. Thus, our study reveals that PKC is able to maintain the homeostasis or viability of *Nf1*-defected cells and may serve as a potential target for developing new therapeutic strategies.

## INTRODUCTION

Neurofibromatosis type 1 (NF1) is an autosomal dominant disease. *Nf1* has a high spontaneous mutation rate occurring in approximately 1 in 3000 individuals [1–3]. Nf1 protein is a GTPase-activating protein that promotes the hydrolysis of Ras from the GTP-bound, active state to GDP-bound form [4–6]. This protein is expressed in various soft tissues and neuronal system [7, 8]. In response to Nf1 defects, Ras activity in the cells is abnormally increased, leading to the promotion of cell proliferation and tumorigenesis. Although NF1 is the most common disease, approximately 10% of the individuals with aberrant *Nf1* develop MPNSTs (malignant peripheral nerve sheath tumors) [9, 10].

Ras family proteins possess GTPase activity and play critical roles in the regulation of various biological processes [11, 12]. Upon mitogenic stimulation, Ras interacts with its downstream effectors, resulting in changes of the phosphorylation status of transcriptional factors and further cell faith [11–14]. Studies demonstrated that cells harboring oncogenic ras were susceptible to cell death when PKC (protein kinase C) was inhibited [15–17]. The link between PKC and Nf1 function was observed [18]. In particular, *Nf1*-defective cells were highly sensitive to PKC inhibitors [19–21]. It was also showed that PKC phosphorylated Ras-like proteins (Rals) and further regulated their subcellular localizations [22]. *Ral* *A* and *B* are expressed in mammalian cells and share 80% homology. Despite the high structural similarity, the functional differences between these two Ral proteins exist, depending upon their interactions with the same or different downstream effectors [23–25]. Ral A appeared to bind to ZO-1-associated nucleic acid binding protein (ZONAB) and Ral binding protein 1 (RLIP76/RalBP1) [26, 27]. Ral B was shown to associate with RLIP76/RalBP1 and SEC5 subunit of exocysts for enhancing the host defense response [28, 29].

RLIP76/RalBP1 possesses two ATP binding sites and functions as an ATP-dependent transporter to control efflux of small molecules [30]. Via influencing activing receptor-interacting protein 2 (ARIP2), RLIP76/RalBP1 upregulated heat shock factor 1 (HSF1) and further influenced the expressions of various factors, including cyclin B1 [31, 32]. Deregulated RLIP76/RalBP1 was detected at the centrosome and on spindle microtubules, accompanied with the occurrence of mitotic catastrophe [32, 33]. Mammalian proteins that are down- or up-stream of Ral have orthologs in *Drosophila* and possess similar functions [34]. The study showed that the *Drosophila* ortholog D-RLIP was bound to the active form of the cyclin B1-p34cdc2 complex, and regulated endocytosis during mitosis [35].

In response to cellular or DNA damage, cell cycle checkpoints function to repair or eliminate injured cells. Two major and partially overlapping pathways regulated by Chk1 and Chk2, play critical roles during repairing damages [36, 37]. Mitotic catastrophe is induced when cells fail a proper division or are unable to enter next cell cycle [38–40]. In late stages of the mitotic phase, cyclin B1 must be degraded in time to allow cells entering next cell cycle [41, 42]. The regulators of the G_2_/M phases are being activated, sometimes in a Chk1-dependent matter [38, 39]. In MPNST cells, a high frequency of mitotic index was often detected [43]. The suppression of PKC triggered a persistently mitotic arrest in *Nf1* deficient cells, which led to a mitotic catastrophe [20, 21]. In this process, Chk1 was phosphorylated and cyclin B1 expression was upregulated. However, the underlying mechanisms by which PKC inhibition triggers mitotic catastrophe in the cells lacking a functional *Nf1* remain unclear.

In this study, we demonstrated that Ral A, but not Ral B, was aberrantly elevated in *Nf1*-defected cells under normal growth conditions. In response to HMG treatment, Ral A was upregulated, accompanied with Chk1 translocation to the nucleus. Subsequently, the cells persistently accumulated in the mitotic phase, resulting in the induction of apoptosis. The animal experiments corroborated well with the *in vitro* findings. Thus, our study suggests that targeting PKC can be a strategy for developing new treatments for NF1-related diseases, especially MPNST patients.

## RESULTS

### PKC inhibition triggers apoptosis via inducing a persistent mitotic arrest under *Nf1*-deficient condition

**Figure 1:**
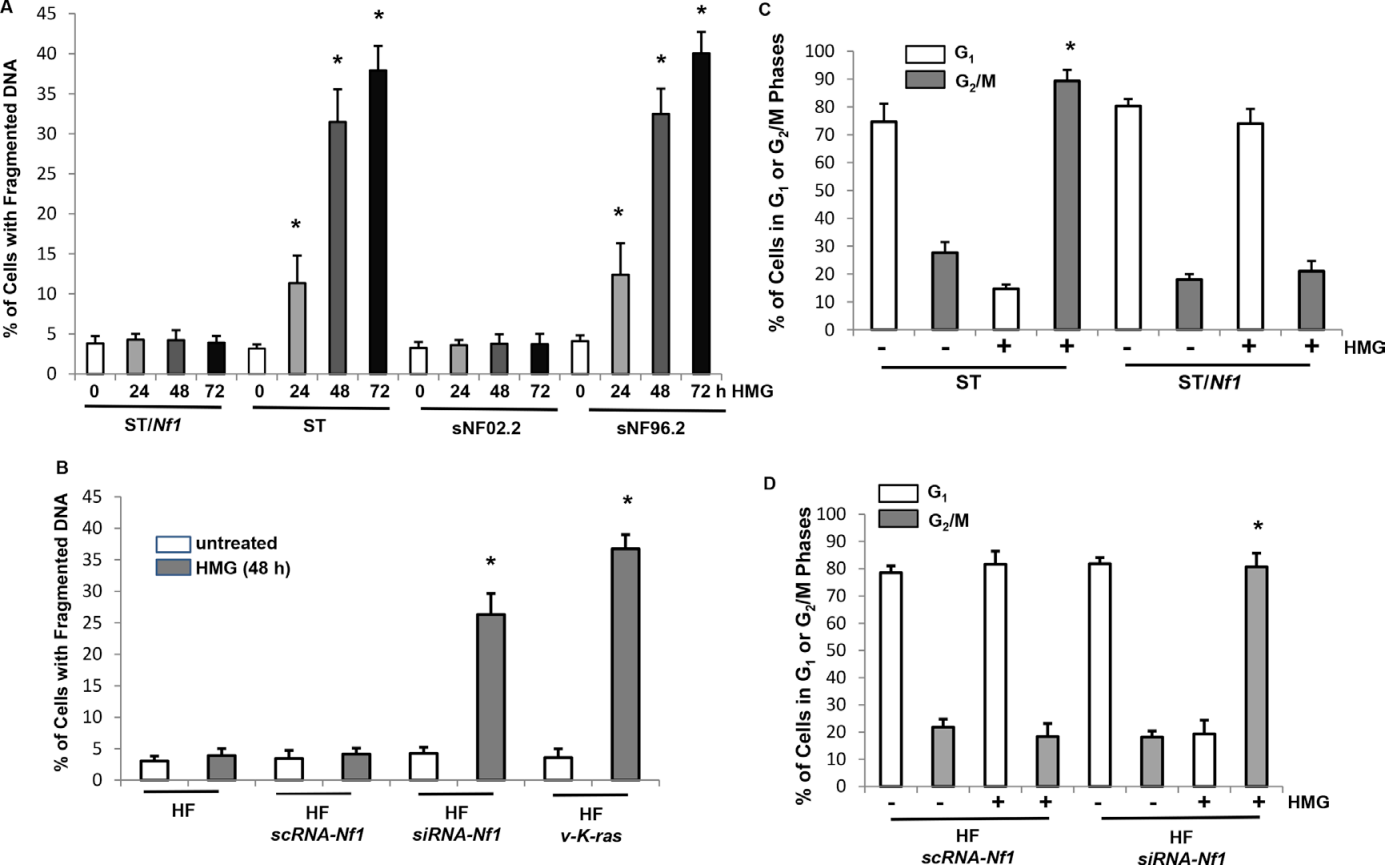
Effects of HMG on the induction of apoptosis and cell cycle progression in *Nf1*-deficient cells treated with HMG (**A** and **B**) *Nf1*-proficient and -deficient cells (A) or human fibroblasts (B) with different genetic manipulations were treated with HMG (10 uM) for various times as indicated. DNA fragmentation assay was then performed. The error bars represent the standard deviation (SD) over 5 independent experiments (*n* = 5, *p < 0.01). (**C** and **D**) Cells were treated with HMG for 24 h, and then subjected to cell cycle analysis, using a flow cytometer. The percentages of the cells accumulated in the G_1_ or G_2_/M phase were measured and plotted. The error bars represent SD from 5 independent experiments (*n* = 5, *p < 0.05).

Cells expressing oncogenic Ras are extremely sensitive to PKC inhibition [15–17]. Since Ras is aberrantly hyperactive in *Nf1*-deficient cells, it led us to test the sensitivity of the cells to PKC inhibition. The induction of apoptosis in *Nf1*-deficient ST, sNF96.2 or proficient sNF02.2, ST/*Nf1* cells (ST cells ectopically express *Nf1* effective domain gene), after HMG treatment at various time points was analyzed by DNA fragmentation assay (Figure 1A). ST and sNF96.2 cells started to undergo apoptosis at 24 h after the addition of the inhibitor, which became evident at 48 h and 72 h of the treatment. In contrast, HMG did not affect the viability of ST/*Nf1* or sNF02.2 cells. The susceptibility of immortalized human fibroblasts (HF) to apoptosis, after the knockdown of *Nf1*, was also examined (Figure 1B). Similar results were obtained. HF cells with knockdown of *Nf1* rapidly underwent apoptosis upon HMG treatment, which did not occur in the control HF cells. In addition, the induction of apoptosis was tested in HF cells overexpressing *v-K-ras*. These cells were also sensitive to PKC inhibition, which is in a good agreement with existing knowledge [15–17]. Furthermore, the phenomenon was confirmed in murine Swiss3T3 fibroblasts ectopically expressing *v-K-ras* (SK1 cells) (Supplementary Figure S1).

During performing DNA fragment analysis, we noticed that a relatively high fraction of *Nf1*-deficient cells resided in the mitotic (M) phase of the cell cycle before undergoing apoptosis. This led us to analyze the cell cycle of ST and ST/*Nf1* cells (Figure 1C and Supplementary Figure S2A, upper panel) or HF cells with or without knockdown of *Nf1* (Figure 1D and Supplementary Figure S2A, lower panel). The majority of ST cells or HF cells lacking *Nf1* accumulated in the M phase 24 h after HMG treatment, which was persistent until 36 h of the treatment (data not shown). However, ST/*Nf1* or HF cells continued to progress in the cell cycle in response to the same treatment. Similar DNA profiles were observed in sNF96.2 and sNF02.2 cells with or without HMG treatment (data not shown). The cell cycle analysis was also performed in HF or Swiss3T3 cells with or without overexpressing *v-K-ras* after HMG treatment (Supplementary Figure S2B and S2C). No M phase arrest was detected in untreated or HMG-treated cells, regardless of ectopic expression of *v-K-ras*. Thus, it indicated although both *Nf1* deficiency and overexpression *v-K-ras* were able to induce apoptosis in the absence of PKC, different mechanisms are being employed.

### Ral A, but not Ral B, is upregulated and binds to Ral/BP1 in *Nf1*-deficient cells after HMG treatment

**Figure 2:**
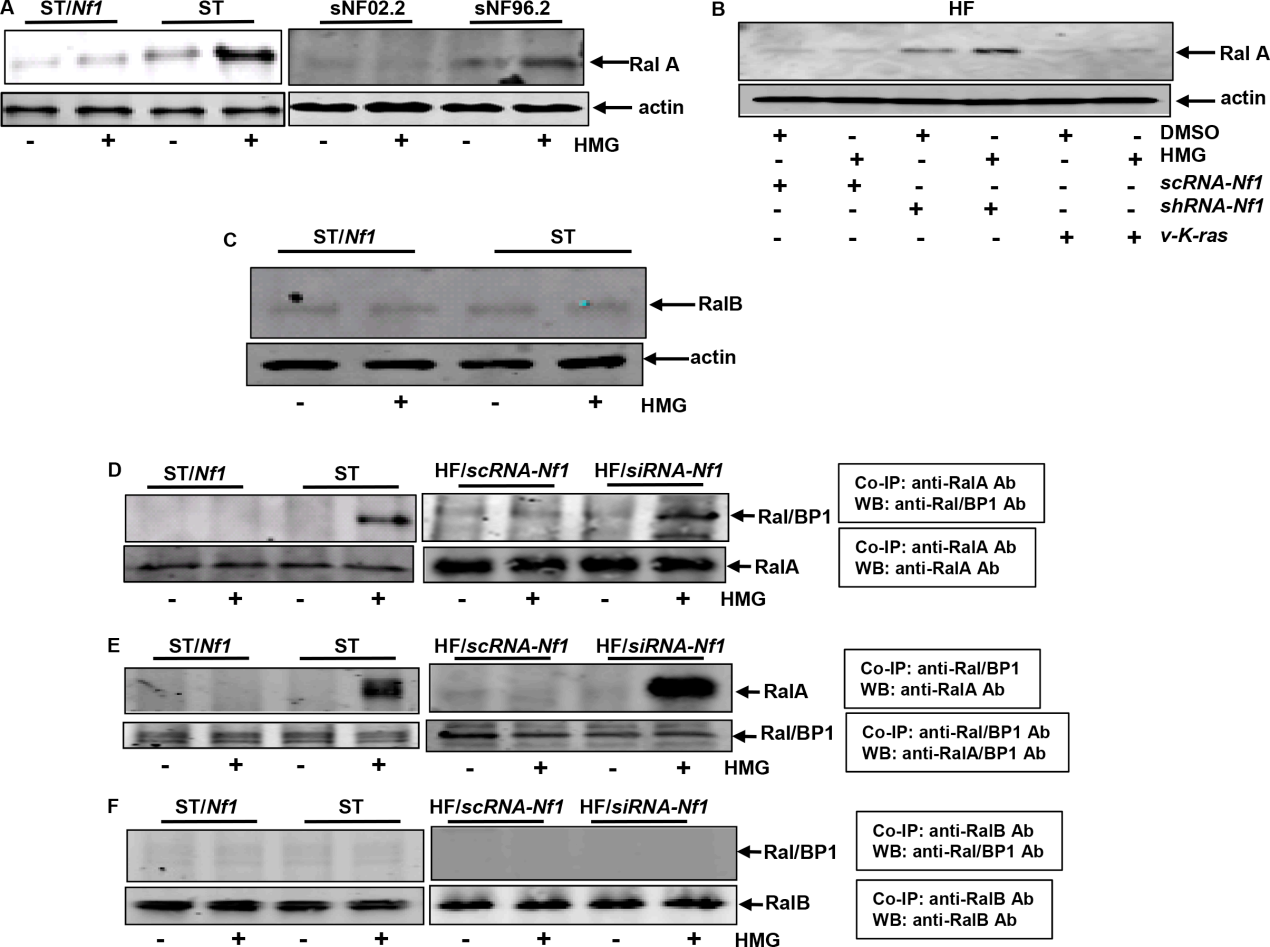
Ral A activation and association with Ral/BP1 in HMG-treated *Nf1*-deficient or -knockdown cells (**A**) Following HMG treatment, the *Nf1*-proficient or -deficient cell lysates were extracted and Ral A activity was measured. The even loadings were normalized by running another immunoblot gel with equal amount of total proteins per lane, which were then blotted with anti-β-actin antibody. (**B**) Ral A activity was analyzed in HF cells with or without the knockdown of *Nf1* and ectopically expressing *v-K-ras* as described above. (**C**) Cell lysates were extracted from untreated or HMG-treated ST or ST/*Nf1* cells as described above. Ral B activity was then examined. (**D**) After HMG treatment, cell lysates were prepared and immunoprecipitated with anti-Ral A antibody. Immunoprecipitates were then blotted with anti-Ral/BP1 antibody. The even loadings of total proteins were normalized by Ral A expression. (**E**) Reciprocal co-immunoprecipitation with anti-Ral/BP1 antibody and immunoblotting with anti-Ral A antibody. The even loadings of total proteins were normalized by Ral/BP1 expression. (**F**) Co-immunoprecipitation with anti-Ral B antibody and immunoblotting with anti-Ral/BP1 antibody were conducted. The even loadings of total proteins were normalized by Ral B expression.

Ral is crucial in the promotion of the growth of MPNST cells [24]. Therefore, we tested the activation status of RalA or B. The activities of Ral A and B in the cells with or without HMG treatment were analyzed using Ral A activation assay kit (Figure 2A). Ral A activity was detectable in *Nf1-*deficient ST or sNF96.2 cells under normal growth conditions. After HMG treatment, Ral A was significantly activated in the deficient cells. The consistent results were obtained from HF cells after knocking-down of *Nf1*, in which Ral A activity was moderately increased and dramatically upregulated upon HMG treatment (Figure 2B). Interestingly, ectopic expression of *v-K-ras* did not activate Ral A, regardless of HMG treatment. Ral B activation was also tested (Figure 2C). HMG treatment did not affect Ral B activity in ST or ST/*Nf1* cells, which was also undetectable in HMG-treated HF/*K-ras* cells (data not shown). In addition, PKC status in *Nf1*-deficient and -proficient cells was tested and this kinase appeared functional in response to its activator PMA (phorbol 12-myristate 13-acetate) or HMG in all of the cells (Supplementary Figure S3A). ERK1 and 2 in *Nf1*-defective cells were phosphorylated, which was not further increase by HMG (Supplementary Figure S3B). The results suggest that Ral A plays a crucial role in apoptosis in HMG-treated *Nf1*-defective cells and determines the divergence of the apoptotic process from that occurs in cells ectopically expressing *v-K-ras*.

Ral A and B are able to bind to a common effector Ral/BP1, but control distinct and sometimes opposing cellular activities [23–25]. To determine whether Rals associate with Ral/BP1 in our experimental setting, co-immunoprecipitation with anti-Ral A antibody and immunoblotting with anti-Ral/BP1 antibody were performed (Figure 2D). The interaction between Ral A and Ral/BP1 was observed only in HMG-treated ST or HF cells after *Nf1* knockdown, which did not occur in the control cells. The reciprocal co-immunoprecipitation and immunoblotting were also conducted (Figure 2E). The same binding patterns were obtained, in which Ral A associated with Ral/BP1 in HMG-treated ST or HF cells after *Nf1* knockdown. The binding of Ral B and Ral/BP1 were also examined (Figure 2F). Ral B did not bind to Ral/BP1 in any cases. The results indicate that Ral A is activated by HMG treatment under *Nf1*-defective condition.

### Chk1 is phosphorylated and translocated to the nucleus in *Nf1*-defective cells treated with HMG

**Figure 3:**
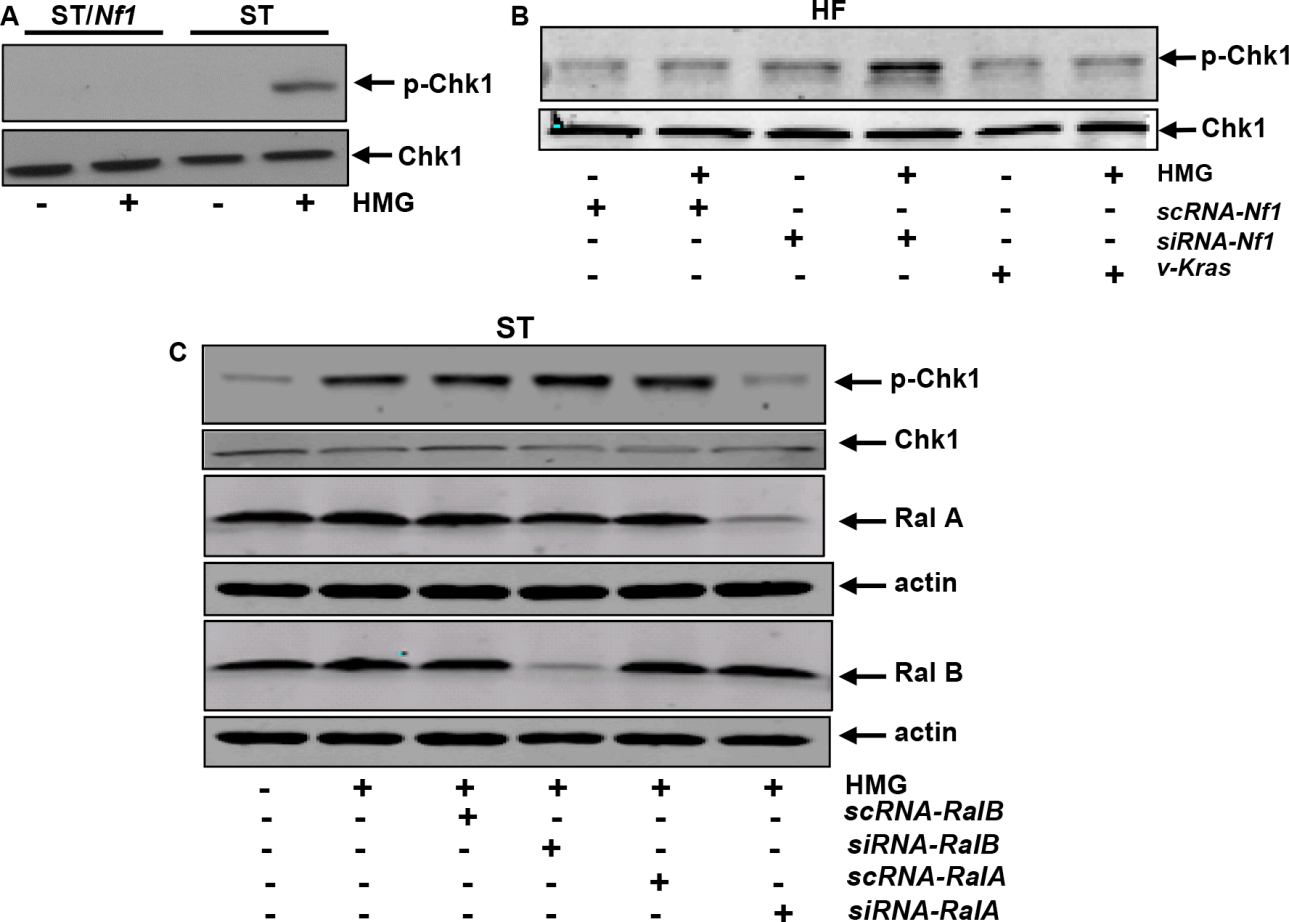
Activation of Chk1 in *Nf1*-deficient or -knockdown cells treated with HMG (**A**) Following HMG treatment, ST or ST/*Nf1* cells were immunoblotted with the anti-phosphorylated-Chk1 antibody. The even loadings of each lanes were normalized by Chk1 expression. (**B**) After transient transfection of the *sc* or *siRN-Rals*, the expressions of p-Chk1, Ral A and Ral B were analyzed in the cells with or without HMG treatment by immunoblotting. The even loadings of total proteins were normalized by β-actin expression. (**C**) Chk1 phosphorylation was performed in HF cells with *Nf1* knockdown or ectopically expressing *v-K-ras*. Even loadings of each lane were normalized by *β*-actin expression.

Cell cycle checkpoints play key roles in eliminating cells with damaged genomes. Checkpoint kinase 1 (Chk1) is one of the important checkpoint regulators and functions to control a cell progressing through each phase of the cell cycle (36, 37). Chk1 activation was accordingly examined in ST or ST/*Nf1* cells with or without HMG treatment (Figure 3A). Chk1 was phosphorylated in response to HMG treatment in ST cells. The phosphorylation of Chk1 was also determined in HF cells transfected with scramble (*sc*), *siRNA-Nf1* or *v-K-ras* in the presence or absence of HMG (Figure 3B). The phosphorylated Chk1 was detected in HMG-treated HF cells after the knockdown of *Nf1*. However, no Chk1 phosphorylation was observed in cells overexpressing *v-K-ras* after adding HMG. We further determined the dependency of Rals on Chk1 activation (Figure 3C). After introduced *siRNA-Ral A* into ST cells, Chk1 was unable to be phosphorylated in HMG-treated ST cells. The knockdown of *Ral B* did not affect Chk1 phosphorylation status, as expected. Overall, It appears that Chk1 functions downstream of RalA in this apoptotic process.

**Figure 4:**
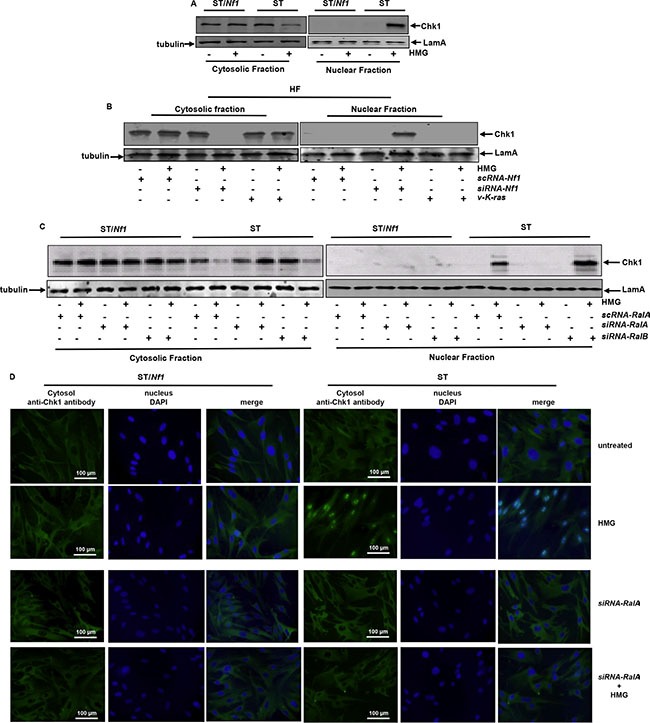
Nuclear translocation of Chk1 in *Nf1* defective cells after HMG treatment (**A**) Cells were treated with HMG and the cytosolic or nuclear fractions were prepared. Subsequently, immunoblotting were conducted, using anti-Chk1 antibody. The even loading of the cytosolic samples was normalized by tubulin and of the nuclear proteins by LamA. (**B**) After knockdown of *Nf1* or ectopically expressing *v-K-ras* in HF cells, Chk1 expression in the cytosolic or nuclear fraction was examined. The even loading of the cytosolic samples was normalized by tubulin and of the nuclear proteins by LamA. (**C**) After the knockdown of Ral A or B, Chk1 expression was tested in each subcellular fraction as described above. (**D**) Immunofluorescent staining of Chk1 in untreated or HMG-treated ST/*Nf1* or ST cells, with or without the transfection of s*iRNA-Ral* A was performed. The slides mounted with the samples were incubated with an anti-Chk1 antibody, and then subjected to the second anti-mouse-IG antibody conjugated with fluorescein and stained with DAPI.

Chk1 possesses a NLS (nuclear localization sequence) at its protein motif 260–271 AA (amino acid) (44, 45). The nuclear translocation of Chk1 is an important feature for its ability to regulate responses triggered by cellular or genetic damages. To further determine Chk1 activation, its nuclear translocation was tested (Figure 4A). After HMG treatment, Chk1 in ST/*Nf1* cells was present in the cytosolic fraction and undetectable in the nuclear fraction. Chk1 in untreated ST cells was mainly in the cytosolic fraction. After HMG treatment, a high amount of Chk1 appeared in the nuclear fraction, which disappeared in the cytosolic fraction. Chk1 nuclear translocation was also tested in HF cells after the knockdown of *Nf1* or ectopic expression of *v-K-ras* (Figure 4B). Consistently, Chk1 was re-located to the nucleus from the cytosol in HMG-treated HF cells without a functional *Nf1*, which could not be seen in HF/*K-ras* cells after the same treatment.

To determine the role of Ral A in the regulation of Chk1 nuclear translocation, *sc,* *siRNA-Ral A* or *B* was introduced into the cells before immunoblotting (Figure 4C). *scRNA-Ral A* or *siRNA-Ral B* did not alter HMG-mediated Chk1 nuclear translocation in ST cells. However, Chk1 nuclear translocation was abrogated by *Ral A* knockdown. To further confirm Chk1 nuclear translocation, immunofluorescence analysis was performed (Figure 4D). The fluorescent staining was mainly seen in the cytosol of untreated or treated ST/*Nf1* cells as well as in untreated ST cells. In comparison, the fluorescein-stained Chk1 was detected in the nucleus of HMG-treated ST cells, which overlapped with DAPI stained nuclei. After the knockdown of *Ral A*, there was no positive Chk1 staining in the nucleus of HMG-treated ST cells. The data suggest that Chk1 is activated in response to HMG treatment under *Nf1*-deficient condition in a Ral A-dependent fashion.

### Ral A is necessary for HMG-mediated M phase arrest and induction of apoptosis in *Nf1*-deficient cells

**Figure 5:**
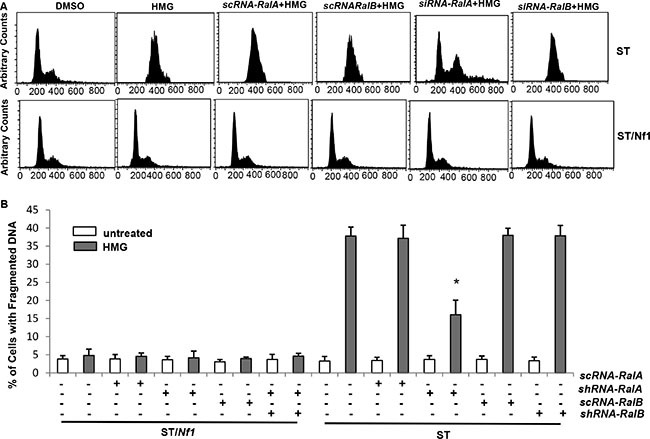
Ral A dependency of HMG-induced mitotic arrest and apoptosis in *Nf1*-deficient cells (**A**) ST or ST/*Nf1* cells were transfected with *scRNA*, *siRNA-Ral A* or *B*, prior to HMG treatment for 24 h, and then cell cycle progression was analyzed, using a flow cytometer. (**B**) After the same treatments as indicated above, DNA fragmentation assay was conducted. The error bars represent SD from 5 independent experiments (*n* = 5, *p < 0.05).

To evaluate the necessity of Ral A for the induction of a persistent M arrest and subsequent apoptosis, *siRNA*-*Ral A* or *Ral B* was transfected into ST/*Nf1* or ST cells, and two portions from each sample were collected for cell cycle (Figure 5A) and apoptotic (Figure 5B) analyses, respectively. The introduction of *scRNA-Ral A* and *sc* or *siRNA-Ral B* did not prevent the majority of HMG-treated ST cells from accumulating in the M phase of the cell cycle. However, after the knockdown of Ral A, the majority of HMG-treated ST cells exited the M phase. DNA fragmentation analysis was in an agreement with the results of cell cycle assay, as the introduction of *siRNA- Ral A* partially blocked the apoptotic process in HMG-treated ST cells. The incomplete blockade of apoptosis by *siRNA-Ral A* suggests the involvement of other factors, other than Ral A, in the regulation of this apoptotic process.

### Apoptosis is induced in xenografted ST tumors upon HMG injection

**Figure 6:**
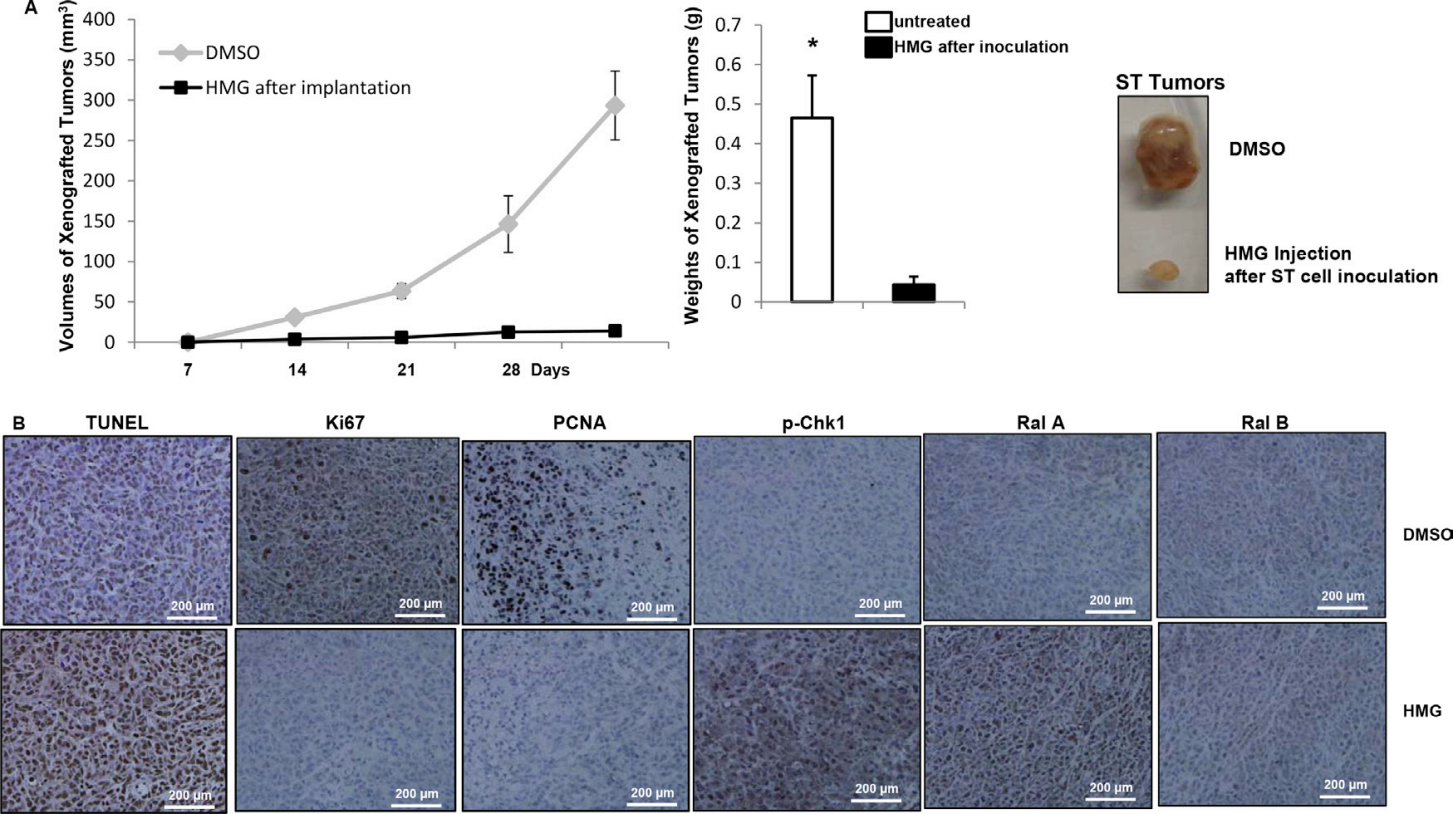
Induction of apoptosis in the xenografted ST tumors or soft tissue tumors isolated from *Nf1*-deficient mice upon HMG injection (**A**) ST cells were inoculated subcutaneously into the nude mice (6 mice per group). HMG was injected peritoneally right after the inoculation and subsequently administrated every 3 days. Once became noticeable, the diameters of the tumors were measured every 7 days and plotted. The error bars represent SD (*n* = 6, *p < 0.05). Twenty-eight days later, the isolated tumors were weighed and data were plotted. The photos of the examples of the tumors were taken. (**B**) The slides mounted with the tumors were stained with TUNEL reagent as well as with anti-Ki67, PCNA, phosphorylated-Chk1, Ral A or Ral B antibody, respectively.

The induction of HMG-induced apoptosis under *Nf1*-deficient condition was further tested by xenograft assay (Figure 6A). After the inoculation of ST cells into nude mice, HMG injection was given every 3 days. The diameters of the tumors in untreated mice were dramatically increased, which were insignificant in the mice injected with HMG (Figure 6A, left panel). Four weeks later, the tumors were isolated and weighed, the results of which were plotted (Figure 6A, middle panel) and picture of which was taken (Figure 6A, right panel). The slides mounted with tumor tissues were stained with terminal deoxynucleotidyl transferase dUTP nick end labeling (TUNEL), or immunohistochemically stained with anti-Ki67, proliferating cell nuclear antigen (PCNA), phor-Chk1, Ral A or Ral B antibody (Figure 6B). The xenografted tumor samples from HMG injected mice showed strong staining with TUNEL, but were negative for the growth marker of Ki67 or PCNA. Consistently, the samples from HMG injected tumors were positively stained with the anti-phor-Chk1 or anti-Ral A antibody, but not with anti-Ral B antibody. In comparison, the pattern of the staining of untreated tumor samples was the opposite. Overall, the results from the *in vivo* experiments and the *in vitro* studies suggest that Ral A is in synergy with PKC inhibition to induce apoptosis in tumors harboring mutated or deleted *Nf1*, via activating the M phase checkpoint.

## DISCUSSION

*Nf1* deficiency in neurofibromatosis has been suggested to render an all-encompassing downstream signaling of Ras [1–3]. Among these, Ral activation under *Nf1*-deficient condition appears a key factor in NF1-related tumorigenesis [24]. The genetic suppression of Ral A significantly blocked the proliferation and migration of MPNST cells [24]. Studies demonstrated that *Nf1*-deficient cells were very sensitive to PKC inhibitors that elicited apoptosis, perhaps through influencing the proteasomal pathway or activating mitotic checkpoints [19–21]. In our current investigation, we determined that Ral A functioned downstream of hyper-active Ras and responsible for the induction of a persistent mitotic arrest, which led to apoptosis in HMG-treated *Nf1*-deficient or knockdown cells. In this apoptotic process, Ral A was associated with Ral/BP1, resulting in Chk1 phosphorylation and further nucleus translocation. The knockdown of *Ral A* relieved the *Nf1*-defective cells from prolonged mitotic arrest, and partially reduced the magnitude of apoptosis. The *in vitro* findings corroborate with the results from the xenograft assay. Thus, our findings provide a possible mechanism by which PKC inhibition sensitizes cells lacking a functional *Nf1* to cell death. We hypothesize that, under normal growth conditions, PKC copes with hyper-active Ras to maintain homeostasis in *Nf1*-defective cells. Once PKC is suppressed, the unstable, aberrant Ras activity, via Ral A, activates the mitotic checkpoint for the induction of apoptosis.

Ral A and B share more than 80% sequence homology, but appear to regulate distinct effectors that influence different gene expression patterns and cellular activities [30]. In some cases, Rals play opposing roles in the promotion of cell growth. In pancreatic cancer cells, Ral A was shown to accelerate the growth of xenografted tumors, but Ral B was required for metastasis [30]. In PC12 cells, Ral A and B functioned oppositely to regulate Raf and PI3K signaling [46]. An increase of Ral-GEF activity suppressed neurite growth and further induced cell cycle arrest. In response to Ral activation, Ral/BP1 was phosphorylated, translocated to the nucleus, and interacted with cyclin B in *Drosophila* and mammalian cells, leading to the formation of the cyclin B-cdc2 complex and subsequent mitotic arrest [27, 35]. Although Rals are shown to be able to bind to common downstream effectors, we identified in our experimental setting that Ral A, but not B, was activated and associated with Ral/BP1.

Mutated *ras* is detected in more than 30% of human cancers [11, 12]. Via governing multiple downstream effectors, oncogenic or aberrant Ras promotes tumorigenesis, which is also proven to be the cause of the cancer predisposition of NF1 patients [9–12]. Tumors harboring oncogenic *ras* were highly sensitive to PKC inhibition [15–17]. PKC appeared crucial for maintaining Nf1 stability [18]. In the present study, we demonstrate that cells lacking a functional *Nf1* are as sensitive to PKC inhibitor HMG as the cells overexpressing *v-K-ras*. During the apoptotic process, Ral A in *Nf1*-defective cells is upregulated and the mitotic checkpoint is activated, which are absent in cells overexpressing *v-K-ras*. Since Ral A and B often function oppositely in regulating certain cellular activities [30] and PKC is required for Ral activation [47], we speculate the divergences of the apoptotic process in HMG-treated *Nf1* defective cells from that in the cells overexpressing *v-K-ras* depend upon the different uses of the downstream effectors. Ral A is activated under *Nf1* defective conditions and perhaps, negatively modulated by PKC. PKC inhibition abrogates such negative regulation, which allows the activity of Ral A to be further upregulated, resulting in activation of the mitotic checkpoint and catastrophe.

**Figure 7:**
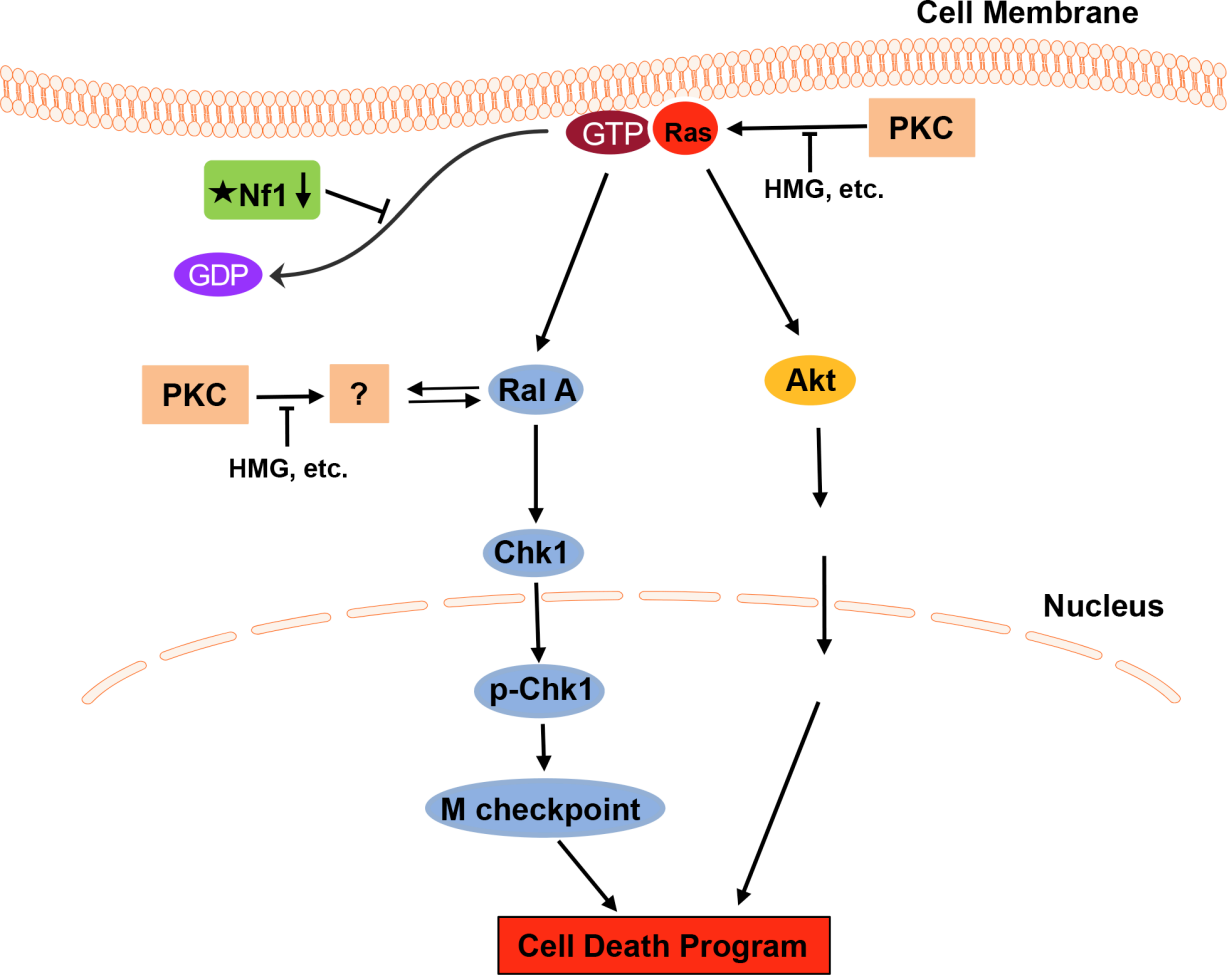
Stochastic signaling pathways for sensitizing *Nf1*-defective cells to apoptosis after PKC inhibition In this apoptotic process, Ral A plays an important role in eliciting a persistent mitotic arrest, which partially contributes to the induction of apoptosis.

Ral activity is linked to the promotion of tumorigenesis, including interfering with the G_2_ checkpoint and cooperating with the epidermal growth factor receptor (EGFR)-mediated signaling for the transformation in rat fibroblasts [30]. In the present study, Ral A was shown to be a key player in the induction of mitotic arrest and subsequent apoptosis in *Nf1*-deficient cells after HMG treatment. Ral A knockdown relieved the defective cells from mitotic arrest, but only partially allows the cells to escape from apoptosis. Previously, we showed that Akt played a role in the induction of apoptosis in *Nf1*-deficient cells after HMG treatment and the suppression of Akt partially blocked this apoptotic process [21]. Together, these data indicate that the apoptotic process elicited by PKC inhibition in *Nf1*-deficient cells is orchestrated by multiple events, including Ral A (Figure 7).

In response to genotoxic stresses (such as DNA damage), cell cycle checkpoints are activated to eliminate damaged cells from being further propagated or prevent injured cells from entering next cell cycle. Such elaborate signaling network operating during DNA damage is mainly regulated by two partially overlapped pathways: Chk1 and Chk2 [36, 37]. A mitotic catastrophe occurs when cells fail to exit from the mitotic phase, which is often regulated by cell cycle checkpoint regulators and one way to eliminate cancerous or diseased cells. In the mitotic phase, DNA damage checkpoint was realized by the phosphorylation and inactivation of Cdk1 or Cdc25C in a Chk1-dependent matter [36, 37]. Here, we further demonstrated that Chk1 was activated and translocated from the cytosol to the nucleus, which triggered a persistent M phase arrest in HMG-treated *Nf1*-deficient cells. We are in the process to elucidate the underlying mechanisms by which Chk1 switches on the mitotic checkpoint for eliminating *Nf1*-deficient cells.

The cooperation between Ras signaling and PKC has been demonstrated [22]. The phorbol ester-dependent PKC isoforms are often involved in pro-survival activities [50]. It is known that different PKC isoforms positively or negatively participate in the regulation of apoptosis induced by geno-toxins and anti-cancer drugs [15, 51, 52]. Under normal growth conditions, PKC activity appears moderately increased in *Nf1*-deficient cells (Supplementary Figure S3A). We speculate that PKC cooperates with or modulates hyper-active Ras signaling to support tumorigenic activities in *Nf1*-deficient cells. Once this balance is disrupted, the hyper-activity of Ras cannot be sustained and an apoptotic crisis is initiated. With increasing attention for searching oncogenic Ras supporting factors or effectors in regulating the vulnerabilities of cancer cells, our study adds the new information for targeting the cooperating molecules (such as PKC) to activate cell death program.

In summary, our study shows that the suppression of PKC specifically induces cells with *Nf1* deficiency to undergo apoptosis, through triggering a persistent M phase arrest. PKC and Nf1 are important signal transducers for cell differentiation and proliferation. Although *Nf1* defect alone is compatible with cell viability, PKC inhibition severely disrupts survival signaling in the cells, which activates a Chk1-dependent, mitotic checkpoint. Because of the lack of effective treatments for preventing and treating NF1-related malignant tumors, our study provides the novel knowledge for developing new therapeutic strategies that can specifically antagonize diseases caused by *Nf1-*deficiency.

## MATERIALS AND METHODS

### Reagents and cell lines

Human *Nf1*-deficient sNF96.2, proficient sNF02.2 cells or human fibroblasts were obtained from ATCC (Manassas, VA) and the details of the phonotypes of the cells were provided by the company. The cells were kept in a −150^0^C freezer after purchasing. The authentications of sNF96.2 and sNF02.2 cells were tested in each study [20, 21]. Human specific *Nf1*-deficient tumor (ST) cells were generous gift from Dr. D Lowy at NCI in 2007 and authenticated by the upregulation of Ras or ERK1/2 as described previously [20, 21]. The cells were cultured in Dulbecco's Modified Eagles's medium supplemented with 10% heat-inactivated fetal bovine serum (Atlanta Biologicals, Flowery Branch, GA), 100 units/ml penicillin, 100 μg/ml Streptomycin (Invitrogen, CA). ST/*Nf1* cells were ST cells stably transfected with the *Nf1* effective domain gene, and maintained in the growth medium containing 200 μg/ml of G418 (Fisher Scientific Inc. MA). HMG was purchased from Santa Cruz Biotechnology (Santa Cruz, CA) and dissolved in DMSO. The control cells represent as the cells growing in the medium containing equal amount DMSO used for dissolving HMG or other inhibitors. Antibodies were purchased from BD (San Jose, CA), Santa Cruz Biotechnology (Santa Cruz, CA), Cell Signaling Technology (Dancers, MA) or Abcam (Cambridge, MA). The *siRNAs* were purchased from Origene (Rockville, MD).

### Flow cytometry analysis

Cell cycle progression and DNA fragmentation data were collected by a Muse Cell Analyzer, and analyzed by the Muse software program (BD Biosciences, Franklin Lakes, NJ). Following treatments, cells were harvested and fixed in 70% cold ethanol. Afterwards, cells were stained with 0.1 μg/ml propidium iodide containing 1.5 ng/ml RNase. DNA profiles of cells were then tested.

### Ral activation assay

Ral activation assay was performed according to the manufacturer's instructions (Cell Biolabs, San Diego, CA). After the treatments, cell lysates were extracted. Half of the lysates were immunoprecipitated with the RalBP1 PBD agarose beads conjugated with the purified protein derivative. Afterwards, the beads bound to active form of Ral A or B were subjected to immunoblotting, using anti-Ral A and anti-Ral B antibodies. Another half of the lysates were immunoblotted with anti-actin antibody for loading controls.

### Co-immunoprecipitation and immunoblot analyses

After the treatments, cell lysates were prepared and immunoprecipitated with an antibody. Subsequently, immunoprecipitates were separated on a SDS-PAGE gel and visualized by Odyssey infrared imaging system (Li-COR Biosciences, Lincoln, NE).

### Preparation of the cytosolic and nuclear fractions

The nuclear and cytosolic fractions were isolated by using the NE-PER nuclear and cytoplasmic extraction kit (Thermo Fisher Scientific, Cambridge, MA). Briefly, ice-cold CER I solution (Cytosolic Extraction Reagent I) was mixed with cell pellets and then added CER II solution (Cytosolic Extraction Reagent II). After centrifugation, the supernatant was collected as cytoplasmic extracts. Cell pellets were re-suspended in the NER (Nuclear Extraction Reagent) and then subjected to centrifugation. The supernatants were collected as the nuclear fractions.

### PKC activity assay

After treatments, PKC activity in cells was analyzed according to the protocol provided by Abcam (Cambridge, UK).

### Immunofluorescent staining

Cells fixed on slides were fixed with 3.7% paraformaldehyde. Following permeabilization with 0.2% TritonX-100, cells were incubated with anti-Chk1 primary antibody (Santa Cruz Biotechnology, CA) and then incubated with Alexa Flour-488 anti-mouse seconday antibody (Invitrogen, CA). The cells were visualized with Zeiss Axio Imager Z microscope. The images were captured using the AxioVision Rel. 4.6 software (Carl Zeiss MicroImaging, NY).

### Xenograft assay

ST cells (5 × 10^6^) in 100 μl of PBS were inoculated into each BalB/c nude mouse. One group of mice (6 mice/group) was injected peritoneally with HMG (30 mg/kg) right after the inoculation and subsequently administrated the same amount of HMG every 3 days. The sizes of the tumors were measured routinely. Twenty-eight days later, the mice were sacrificed and the tumors were isolated and weighted. The slides mounted with tumor samples were stained with TUNEL staining or subjected to immunohistochemistry analysis with corresponding antibodies.

All the animal experiments were carried out according to the guidelines of the Animal Care and Use Committees of the Institute.

### Statistical analysis

Statistical analysis was performed using a two-tailed Student's *t* test for comparison of two groups or a one-way analysis of variance for comparison of more than two groups followed by Tukey's multiple comparison tests. Tumor-free probabilities were estimated using Kaplan-Meier method and were compared among groups. Standard deviations are displayed in the figures. A *p value* < 0.05 was considered significant.

## SUPPLEMENTARY MATERIALS FIGURES AND TABLES


