# Supplementary material for: Ral A, via activating the mitotic checkpoint, sensitizes cells lacking a functional Nf1 to apoptosis in the absence of protein kinase C
Source: Oncotarget. 2016 Oct 12;7(51):84326–37. doi: 10.18632/oncotarget.12607 (PMC5356664; doi:10.18632/oncotarget.12607)
Supplement: Supplementary file 1 [file oncotarget-07-84326-s001.pdf]

Ral A, via activating the mitotic checkpoint, sensitizes cells lacking a functional *Nf1* to apoptosis in the absence of protein kinase C

Supplementary Materials

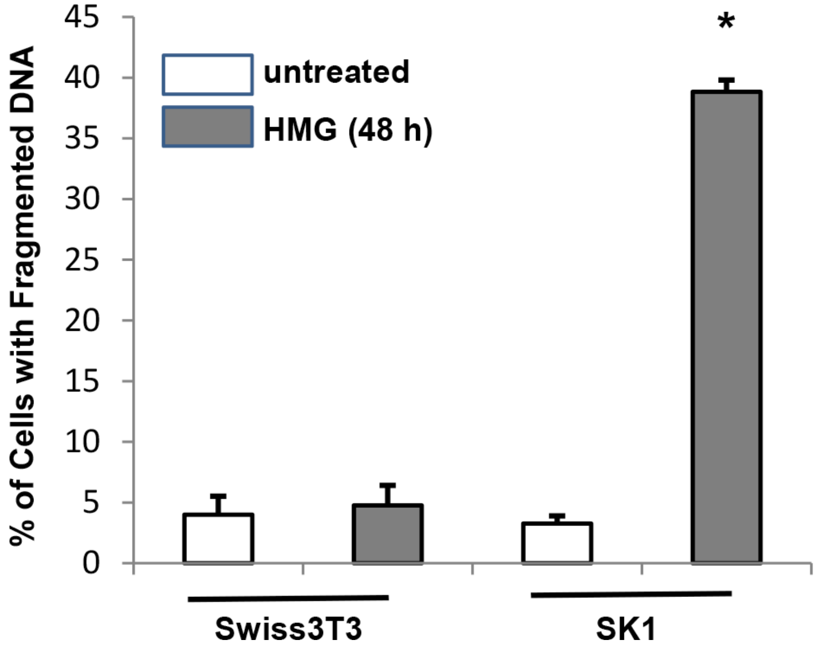

Supplementary Figure S1: Effects of HMG on the induction of apoptosis in the cells ectopically expressing *v-K-ras*. Swiss3T3 cells or *v-K-ras* trasnfectants (SK) were treated with HMG for 48 h. DNA fragmentation assay was then performed. The error bars represent the standard deviation (SD) over 5 independent experiments ($n = 5$, *$p < 0.01$).

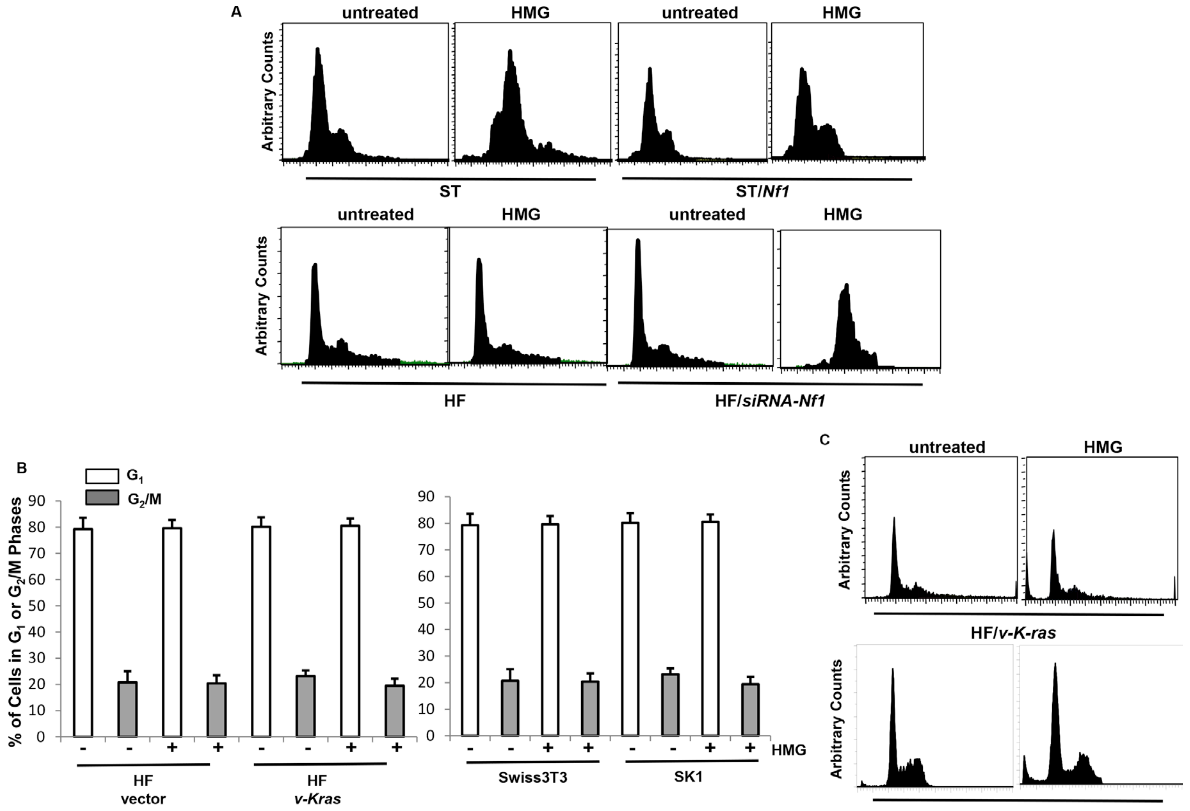

Supplementary Figure S2: M phase accumulation in HMG-treated cells lacking a functional *Nf1*. (**A**) DNA profiles in the cells with or without expressing a functional *Nf1*, following HMG treatment, were analyzed. (**B**) DNA profiles in the cells overexpressing *v-K-ras* were determined with or without HMG treatment and percentages of the cells in different phases were plotted. The error bars represent the standard deviation (SD) over 5 independent experiments ($n = 5$, $*p < 0.005$). (**C**) DNA profiles in the cells with or without ectopic expressing a *v-K-ras* were analyzed following HMG treatment.

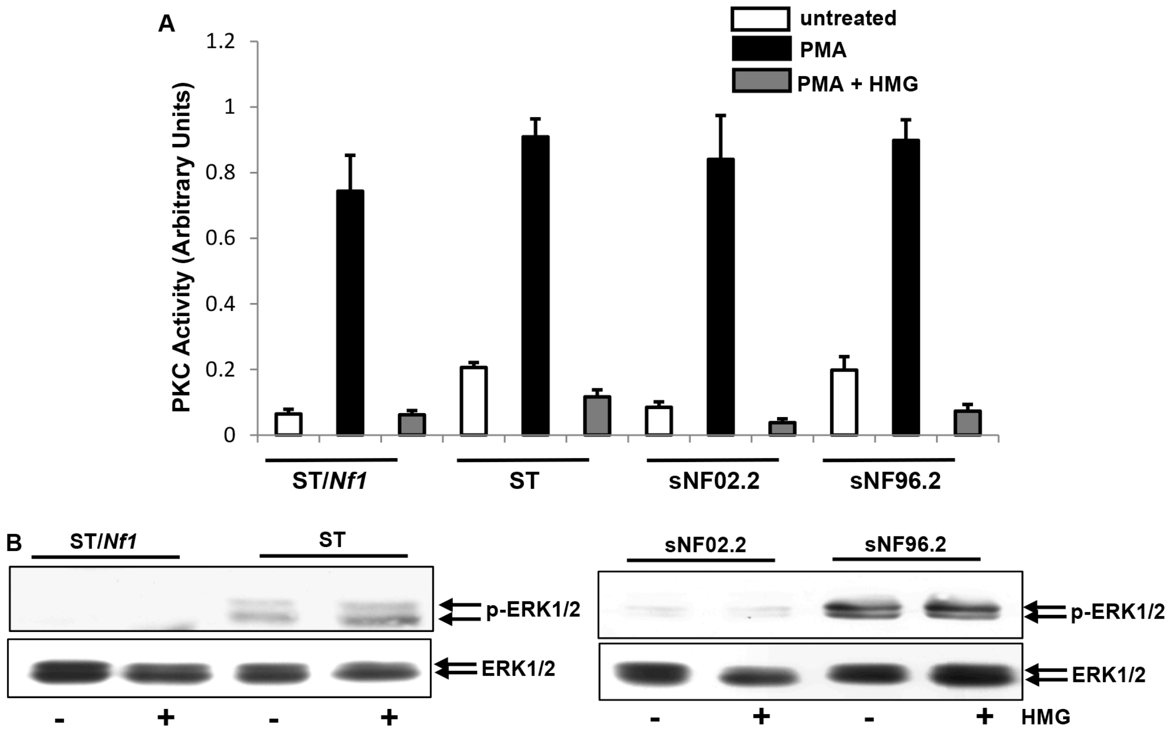

Supplementary Figure S3: PKC activity and ERK1/2 activation in *Nf1*-deficient cells. (**A**) Cell lysates isolated from ST, ST/*Nf1*, sNF02.2 and sNF96.2 cells with or without being treated with PMA or PMA plus HMG were isolated and tested with PKC kinase activity kit. The error bars represented SD over 5 independent experiments ($n = 5$, $*\rho < 0.05$). (**B**) After HMG treatment, cell lysates were prepared and then immunoblotted with the anti-phosphorylated ERK1/2 antibody. The even loadings of total proteins were normalized by ERK1/2 expression. The even loadings of the lanes were normalized by β-actin expression.
